# Integrative modeling identifies genetic ancestry-associated molecular correlates in human cancer

**DOI:** 10.1016/j.xpro.2021.100483

**Published:** 2021-04-19

**Authors:** A. Gordon Robertson, Christina Yau, Jian Carrot-Zhang, Jeffrey S. Damrauer, Theo A. Knijnenburg, Nyasha Chambwe, Katherine A. Hoadley, Anab Kemal, Jean C. Zenklusen, Andrew D. Cherniack, Rameen Beroukhim, Wanding Zhou

**Affiliations:** 1 Canada’s Michael Smith Genome Sciences Centre, BC Cancer, Vancouver, BC V5Z 4S6, Canada; 2 Buck Institute for Research on Aging, Novato, CA 94945, USA; 3 Department of Surgery, University of California, San Francisco, San Francisco, CA 94115, USA; 4 The Broad Institute of Harvard and MIT, Cambridge, MA 02142, USA; 5 Department of Medical Oncology, Dana-Farber Cancer Institute, Boston, MA 02215, USA; 6 Harvard Medical School, Boston, MA 02115, USA; 7 Lineberger Comprehensive Cancer Center, University of North Carolina at Chapel Hill, Chapel Hill, NC 27599, USA; 8 Institute for Systems Biology, Seattle, WA 98109, USA; 9 National Cancer Institute, Bethesda, MD 20892, USA; 10 Department of Medicine, Brigham and Women’s Hospital, Boston, MA 02115, USA; 11 Department of Cancer Biology, Dana-Farber Cancer Institute, Boston, MA 02215, USA; 12 Center for Computational and Genomic Medicine, Children’s Hospital of Philadelphia, Philadelphia, PA 19104, USA; 13 Department of Pathology and Laboratory Medicine, University of Pennsylvania, Philadelphia, PA 19104, USA

**Keywords:** Bioinformatics, Cancer, Genomics

## Abstract

Cellular and molecular aberrations contribute to the disparity of human cancer incidence and etiology between ancestry groups. Multiomics profiling in The Cancer Genome Atlas (TCGA) allows for querying of the molecular underpinnings of ancestry-specific discrepancies in human cancer. Here, we provide a protocol for integrative associative analysis of ancestry with molecular correlates, including somatic mutations, DNA methylation, mRNA transcription, miRNA transcription, and pathway activity, using TCGA data. This protocol can be generalized to analyze other cancer cohorts and human diseases.

For complete details on the use and execution of this protocol, please refer to Carrot-Zhang et al. (2020).

## Highlights

• Protocols for ancestry associations with TCGA molecular data
• Protocols for ancestry associations with oncogenic pathways
• Statistical and power analysis for determining significant associations
• Key considerations of potential confounding factors

## Graphical abstract

**Figure:**
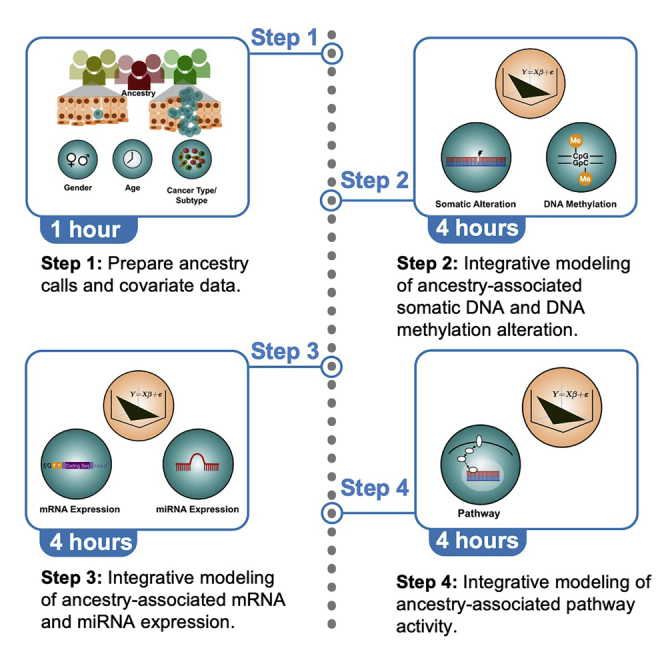

## Before you begin

**Timing: 0.5–1 h**

1. Download ancestry assignments from Table S1 of (Carrot-Zhang et al., 2020) (see key resources table, raw ancestry calls and admixture estimates are available at the GDC publication page https://gdc.cancer.gov/node/1248) . The ancestry calls include patient classification into European (EUR), East Asian (EAS), African (AFR), Native/Latin American, and (South) Asian, using genotyping calls from SNP6.0 array and exome sequencing data (see the companion protocol Carrot-Zhang et al., 2020 for details). This file also indicates admixed individuals and their level of admixture.
2. Prepare cancer types and subtypes by downloading Table S1 of Sanchez Vera et al. 2018 (Sanchez-Vega et al., 2018) (see key resources table). Obtain bladder cancer mRNA subtypes from Table S1 of Robertson et al., 2018, "mRNA_cluster" column (Robertson et al., 2018) (see key resources table). Consolidate the two into a single subtype table.
3. Download age and gender information from the "clinical with follow up" file from https://gdc.cancer.gov/about-data/publications/pancanatlas (see key resources table). Use "age_at_initial_pathologic_diagnosis" for age.
4. Download the sample quality whitelist from Genomic Data Commons (see key resources table)

**CRITICAL:** Certain cancer types are heterogeneous, and one can further classify those cancer types into various subtypes. This heterogeneity can significantly confound ancestry association when specific ancestries are overrepresented in certain subtypes. For example, for bladder cancer, East Asian ancestry is enriched in the luminal papillary subtype (Robertson et al., 2018). Targeting the ancestry association of bladder cancer without resolving subtypes often leads to detection of molecular correlates that are associated with different subtypes rather than ancestry alone (see expected outcomes).

***Alternatives:*** This protocol uses TCGA subtype information, which exists for only 12 of the 33 cancer types. Alternatively, one can also use iCluster classification (Hoadley et al., 2018) which also classifies cancers based on molecular characteristics and captures shared features across cancer types.

## Key resources table

**Table:** 

REAGENT or RESOURCE	SOURCE	IDENTIFIER
**Deposited data**		

TCGA patient ancestry assignment	Carrot-Zhang et al., 2020*Cancer Cell*	Table S1 of 10.1016/j.ccell.2020.04.012
TCGA cancer type and subtype	Sanchez Vera et al., 2018*Cell*	https://ars.els-cdn.com/content/image/1-s2.0-S0092867418303593-mmc1.xlsx
TCGA BLCA mRNA subtype	Robertson et al., 2018*Cell*	https://ars.els-cdn.com/content/image/1-s2.0-S0092867417310565-mmc1.xlsx
TCGA patient gender and age	Liu *et al.* 2018 *Cell* (Liu et al., 2018)	https://api.gdc.cancer.gov/data/1b5f413e-a8d1-4d10-92eb-7c4ae739ed81
TCGA sample quality whitelist	Genomic Data Commons	http://api.gdc.cancer.gov/data/1a7d7be8-675d-4e60-a105-19d4121bdebf
TCGA MC3 mutation calls	Ellrott et al. 2018 *Cell Systems* (Ellrott et al., 2018)	http://firebrowse.org/api-docs/#!/Analyses
Cancer mutational signature	Alexandrov *et al.* 2013 *Nature* (Alexandrov et al., 2013)	https://www.synapse.org/#!Synapse:syn11801497
TCGA ABSOLUTE-estimated purity	Genomic Data Commons	http://api.gdc.cancer.gov/data/4f277128-f793-4354-a13d-30cc7fe9f6b5
TCGA local ancestry call	Carrot-Zhang et al., 2020*Cancer Cell*, Genomic Data Commons publication page	https://api.gdc.cancer.gov/data/d773a53e-b78d-4a20-848c-866616352328
TCGA Affymetrix SNP 6.0 microarray data	Genomic Data Commons Legacy Archive	https://portal.gdc.cancer.gov/legacy-archive/
TCGA GISTIC copy number call	GISTIC: McCarroll *et al.* 2008 *Nature Genetics* (McCarroll et al., 2008)	http://firebrowse.org/api-docs/#!/Analyses
TCGA chromosome arm calls and aneuploidy scores	Taylor *et al.* 2018 *Cancer Cell* (Taylor et al., 2018)	https://ars.els-cdn.com/content/image/1-s2.0-S1535610818301119-mmc2.xlsx
TCGA Infinium BeadChip data (DNA methylation level, or beta values)	Genomic Data Commons Legacy Archive	https://portal.gdc.cancer.gov/legacy-archive/
Probe annotation for Infinium BeadChip platform	Zhou et al., 2017*Nucleic Acids Res*	http://zwdzwd.github.io/InfiniumAnnotation
TCGA WGBS for 49 samples	Zhou *et al.* 2018 *Nature Genetics* (Zhou et al., 2018a)	http://zwdzwd.github.io/pmd
TCGA normalized mRNA data	Genomic Data Commons, Pancan Atlas Portal	https://gdc.cancer.gov/about-data/publications/pancanatlas
TCGA normalized mature strand miRNA RPM data	Genomic Data Commons, Pancan Atlas Portal	https://gdc.cancer.gov/about-data/publications/pancanatlas
miRNA annotation from miRBase	Kozomara et al., 2019*Nucleic Acids Res*	RRID: SCR_003152
GFF3 for gene annotation	Ensembl v94 gene annotations	https://uswest.ensembl.org/info/data/ftp/index.html see also Ensembl Archives: https://m.ensembl.org/info/website/archives/index.html
GRCh38 cytoband coordinates	UCSC Genome Browser	http://hgdownload.soe.ucsc.edu/goldenPath/hg38/database/cytoBand.txt.gz
General miR-gene resource	Carrot-Zhang et al., 2020*Cancer Cell*	https://gdc.cancer.gov/about-data/publications/CCG-AIM-2020 miRNA_information.xlsx
GFF3 for miRNA stem-loop and mature strand annotations from miRBase	Kozomara *et al.* 2019 *Nucleic Acids Res* (Kozomara et al., 2019)	http://www.mirbase.org/ftp.shtml, and previous releases. hsa.gff3.txt
PARADIGM Pathway Activity Level	Campbell *et al.* 2018 *Cell Reports* (Campbell et al., 2018)	http://api.gdc.cancer.gov/data/7d4c0344-f018-4ab0-949a-09815f483480
PARADIGM Pathway Definition	Campbell *et al.* 2018 *Cell Reports* (Campbell et al., 2018)	Supplemental information Feature_Dictionary_pid_20130518.txt Interaction_Dictionary_pid_20130518.txt

**Software and algorithms**		

PARADIGM for integrative pathway analysis	Vaske *et al.* 2010 *Bioinformatics* (Vaske et al., 2010)	http://sbenz.github.io/Paradigm/
TargetScan for miRNA target detection	Agarwal et al., 2015*eLife*	http://www.targetscan.org/faqs.Release_7.html
SeSAMe for DNA methylation data preprocessing	Zhou et al., 2018b*Nucleic Acids Res*	https://bioconductor.org/packages/release/bioc/html/sesame.html

**Other**		

Resource website for the Ancestry-associated Molecular Correlates	Carrot-Zhang et al., 2020*Cancer Cell*	https://gdc.cancer.gov/about-data/publications/CCG-AIM-2020

## Materials and equipment

### Privacy protection

The analyses outlined in this protocol do not require controlled-access data. All human subjects have been de-identified. However, researchers who study these data should comply with TCGA policies such as maintaining participants' privacy, accessing data securely, and following TCGA publication guidelines.

## Step-by-step method details

### Association of ancestry with somatic alterations: Pan-cancer analysis

**Timing: 2 h**

1. Download significantly mutated gene lists for each cancer type defined by MutSig2 (see MC3 mutation call file in key resources table) (Bailey et al., 2018). Merge them to create a list of significantly mutated genes across cancers.
2. Create a mutation frequency file for each significantly mutated gene by counting the number of samples with one or more single nucleotide variants (SNVs) or small indels (use the MC3 mutation call file, see key resources table) and focal copy number amplifications in that gene (use the pan-cancer GISTIC copy number calls, see key resources table) across all cancer types.

***Note:*** we include different somatic mutation types, including SNVs, indels, and focal amplification (defined as log2 copy number ratio >1)

3. Exclude ancestry-admixed samples (20%<non-EUR ancestry<80%) and genes with less than 30 altered samples in the AFR group.

***Note:*** We chose the threshold for ancestry-admixed samples to ensure we have enough non-European ancestry samples while excluding samples with a high-degree of ancestry mixture to allow a clear comparison between populations. We found two samples with more than 20% ancestry in more than two ancestry groups (three-way admixed individuals) in TCGA. We included those two samples by associating the percentage of each ancestry with genomic alterations.

4. Combine the above file with the TCGA ancestry assignments, clinical data, subtype annotation (key resources table)
5. Code EUR ancestry as 0 and AFR ancestry as 1. Code the somatic alteration status into a binary variable that represents Tumor Mutation Burden (TMB). Samples with one or more mutations have TMB 1, otherwise 0. Perform logistic regression of somatic alteration status on ancestry, controlling for age, gender, and subtype. One could use the following model in R:
  [TMB] ~ [ancestry] + [age] + [gender] + [subtype]
6. Apply Benjamini-Hochberg FDR correction to all the p-values
7. Repeat steps 3–6 to analyze somatic alterations between EUR and EAS by coding EUR as 0 and EAS as 1.
8. Visualize ancestry-associated genes using a Q-Q plot (see Figure 2A in (Carrot-Zhang et al., 2020))
9. Repeat steps 3–8 for each cancer type and perform cancer-type-specific analysis

***Note:*** Ancestry-specific somatic alterations can be tissue-type specific. For certain cancer types, a large set of sample size for non-European samples is needed to reach sufficient detection power (see Problem 1 and power analysis in the quantification and statistical analysis section below).

10. Validate genes identified as ancestry-associated using the admixed individuals excluded from the prior analysis (20%<AFR ancestry<80%). Calculate the Spearman’s correlation between their mutation frequencies and the fraction of AFR or EAS ancestry per individual obtained from ADMIXTURE (Alexander et al., 2009) (see ancestry call file in the key resources table). Use the Wilcoxon rank-sum test to assess statistical significance.
11. Download the mutational signature file (see key resources table). Correlate each mutational signature (SBS1-SBS50) with ancestry, controlling for age, gender, Tumor Mutation Burden (TMB), and subtype, i.e.,
  [mutational signature] ~ [ancestry] + [age] + [gender] + [subtype] + [TMB]
12. Apply the Benjamini-Hochberg FDR correction to all the p-values from the mutation signature correlations.
13. Download TCGA arm-level somatic copy number alteration calls (SCNAs), genome doubling, aneuploidy scores, and leukocyte fraction or immune infiltration scores (IMS) calculated based on methylation (see key resources table). Correlate ancestry with each of these, controlling for age, gender, and cancer subtype, using the following logistic regression model in Python:
  [ancestry] ~ [arm-level SCNA] + [aneuploidy] + [genome doubling] + [age] + [gender] + [subtype]
14. Apply the Benjamini-Hochberg FDR correction to all these p-values.
15. Validate genes with ancestry-specific somatic alterations identified from cancer-type-specific analysis (FDR < 0.1) by comparing alteration frequencies in AFR vs. EUR and EAS vs. EUR using Fisher’s exact test, in an independent cancer cohort (e.g., the Foundation Medicine, FMI Cohort as used in (Carrot-Zhang et al., 2020))

**CRITICAL:** Inclusion of cancer subtype in the TCGA pan-cancer analysis is necessary to eliminate mutations enriched in specific subtypes due to uneven distribution of ancestry in the subtypes (e.g., breast cancer). Other clinical characteristics may also be considered potential confounders in the somatic mutation-ancestry correlations, e.g., disease stage and smoking history.

***Note:*** For validation using external data sets, it is crucial to ensure that the disease ontology terms are harmonized with TCGA data sets. In some cases, external datasets will not have as detailed subtype information. The correlation between somatic alteration frequency and the fraction of AFR or EUR ancestry per individual is performed using binomial logistic regression. It is also important to use the same procedure of ancestry calling for independent cancer cohorts. See our companion protocol Carrot-Zhang et al., 2020, for how to generate ancestry calling from genotyping data.

### Association of ancestry with DNA methylation

**Timing: 3 h**

**Timing: 1 h for step 16**

**Timing: 1 h for step 17**

**Timing: 1 h for step 18**

**Figure 1:**
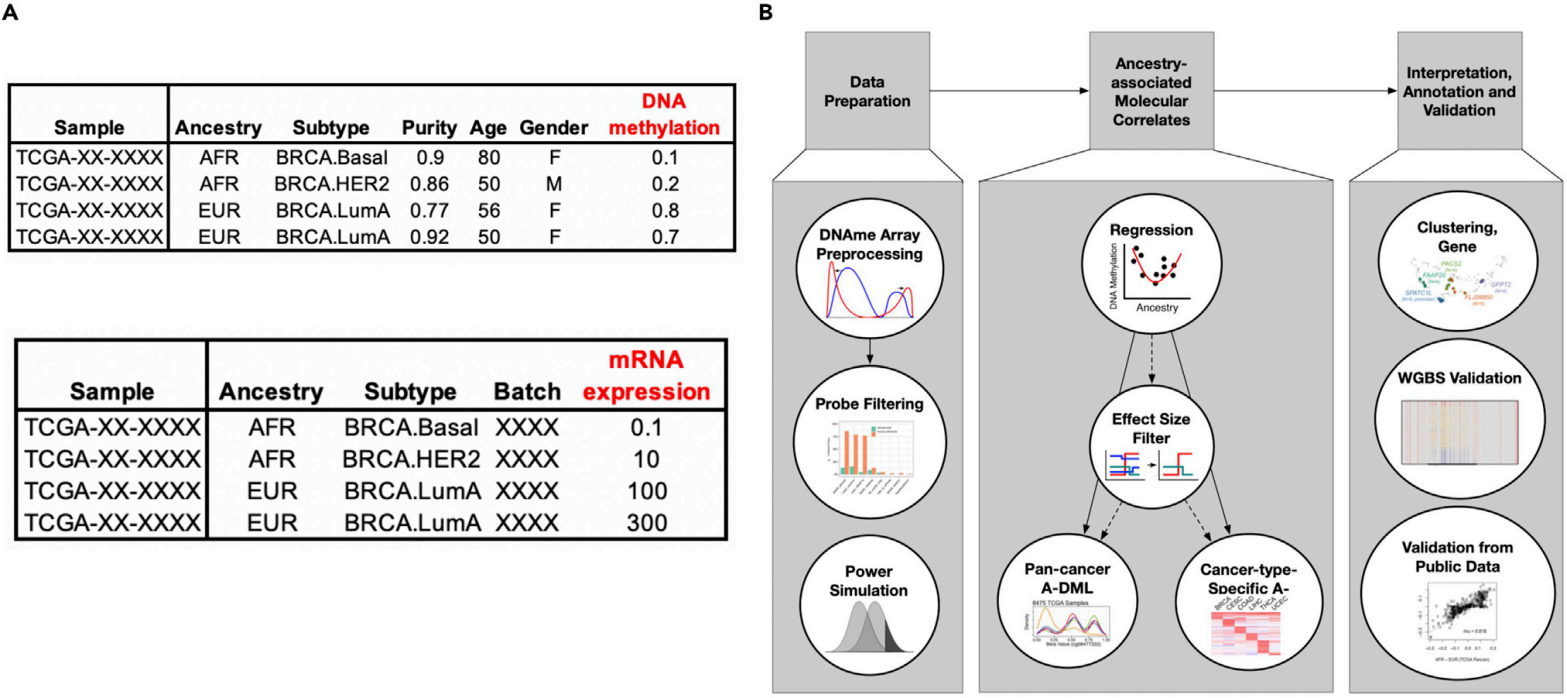
Input format example and workflow for ancestry association (A) Example input files for regression. The response variable is colored in red. Top: DNA methylation. Bottom: mRNA expression. (B) General analysis workflow, showing DNA methylation in expanded panels.

16. Filter Infinium DNA methylation BeadChip probes directly influenced by genetic variation.
  a. Download DNA methylation IDATs from the GDC legacy archive (see key resources table) and process them to beta values using SeSAMe (Zhou et al., 2018b).
    ***Note:*** The obtained beta value file is a masked matrix with NA indicating either lack of signal or design flaws. More detailed information about masking can be found in (Zhou et al., 2017). Some cancer types in the GDC legacy data were not consistently processed. The previous processing pipeline estimated signal background using internal negative controls (Davis et al., 2012). This pipeline underestimated the true background, leading to the potential false-positive discovery of epigenetic silencing (Zhou et al., 2018b).
  b. Download sample annotations (see key resources table), including
    i. Sample quality annotations as reported in the quality annotation whitelist.
    ii. Gender as reported in the clinical data table
    iii. Age as reported in the clinical data table
    iv. ABSOLUTE-estimated purity data. Tumor adjacent normal samples have purity 0.
  c. Filter samples using the pan-cancer whitelist and remove admixed samples. Remove samples of undetermined purity.
    ***Alternatives:*** 625 samples do not have purity annotation. The missing annotation is likely due to a lack of copy number variation signal needed for the ABSOLUTE method (Carter et al., 2012). In this analysis, we remove samples with unknown purity estimates. One can also use other ways to estimate major non-tumor components such as stromal cells and leukocytes to replace purity. These contaminants can be estimated using mRNA or DNA methylation signatures ((Thorsson et al., 2018) provides leukocyte estimates).
  d. Create a matrix file with rows corresponding to the sample and columns corresponding to CpGs, and the sample covariates (see Figure 1 for a schematic example).
    ***Note:*** In R, we need to convert categorical sample covariates to factors. During the model fitting, it is better to fix the reference group for each sample covariate (e.g., use EUR as the reference for ancestry) to stabilize the slope coefficient in different runs.

**CRITICAL:** Ancestry is closely linked to genetic background. Infinium DNA methylation probe hybridization can be influenced by genetic variations that occur in the probe sequence. SNPs on the probe sequence within 5bp from the 3**′**-end strongly impact the hybridization and extension efficiency, leading to biased readouts of signal intensities (Zhou et al., 2017). SNPs that occur at the probe extension base and flip the designated detection color channel can produce false readouts of methylation levels. These generate measurement artifacts which could be erroneously interpreted as ancestry-associated DNA methylation. We recommend excluding CpGs interrogated using Infinium probes with a common SNP found within the last 5 bp from the target CpG and at the probe extension. This information can be downloaded from the Infinium DNA methylation probe annotation database (see key resources table).

17. Test DNA methylation for ancestry association
  a. For each probe, fit two linear regression models, with and without ancestry, while accounting for cancer subtype, gender, age, and tumor purity, i.e.,
    i. Full (with ancestry): [DNA methylation β-value] ~ [ancestry] + [cancer subtype] + [gender] + [purity] + [age]
    ii. Reduced (without ancestry): [DNA methylation β-value] ~ [cancer subtype] + [gender] + [purity] + [age]
  b. Test the F-statistics and p-value for ancestry association by contrasting the two regression models. In R, one can use the *anova(full, reduced, test="F")* function.
  c. Apply the Benjamini-Hochberg FDR correction to all the p-values.
  d. Identify ancestry-associated methylation as CpGs with FDR-corrected q < 0.05. One may optionally filter CpGs with a small effect size. See Problem 2. Effect size is defined as the difference in means between the most and least methylated ancestry groups.
  e. Repeat steps 16c–17c for the seven cancer types with more than ten non-EUR samples (BLCA, BRCA, CESC, COAD, LIHC, THCA, and UCEC).

***Note:*** One can validate the effectiveness of the fitting by inspecting the fitted slope coefficient for cancer subtypes against known biology. For example, in TGCT one will expect an excessive number of CpGs with loss of DNA methylation due to its cell of origin, and in IDH-mutated gliomas one expects a global gain in DNA methylation.

18. Interpret ancestry-associated DNA methylation
  a. For both pan-cancer analysis and cancer type-specific analysis, tally the number of ancestry-associated CpGs.
    ***Note:*** The Infinium HumanMethylation450 BeadChip has 65 built-in SNP probes (probes with ‘rs’ in the probe name). One can use these standard SNP probes as positive controls for ancestry association since these SNPs reflect the genetic background and differ between ancestry groups. For example, in our pan-cancer analysis, 63 of these 65 SNP probes were significantly associated with ancestry (see Figure 3A in (Carrot-Zhang et al., 2020)). One can also use the fraction of SNP probes identified to estimate recall. For example, cancer types with inadequate non-EUR representation have less power in detecting ancestry association, and pan-cancer analyses are more powerful than cancer type-specific analyses (see Figure S3F in (Carrot-Zhang et al., 2020)). The same applies to other confounders to ancestry association such as unaccounted cancer-to-cancer heterogeneity. The effect of these confounders depends on the participating cells and the genomic locations of the CpGs of interest. To mitigate such confounding, one could incorporate a more granular cancer type/subtype classification or perform a pan-cancer analysis where multiple cancer types are pooled to strengthen the signal.
  b. Annotate ancestry-associated CpGs for gene associations using the Probe annotation file (see key resources table). CpG-gene associations are classified as promoters and gene bodies. Promoter CpGs are defined as CpGs located from 1500 bp upstream of the Transcription Start Site (TSS) until the Transcription Termination Site (TTS) of any isoform of the gene.
    ***Note:*** It is essential to use a comprehensive gene model to capture all biotypes. Many CpGs are associated with noncoding RNAs, pseudogene transcription, and endogenous viral elements. Our annotation uses GENCODE v22. One should use a more updated gene model if available. A significant fraction of the ancestry-associated CpGs are not identified as associated with genes. CpGs at potential regulatory enhancer elements can be identified using the latest cis-regulatory element definition, such as (Abascal et al., 2020).
  c. Group CpGs that are associated with the same gene. Cluster and plot the ancestry association slope coefficients.
    ***Note:*** CpGs in genomic proximity tend to undergo coordinated methylation changes, indicating potential local chromatin states linked to gene expression control. This spatial correlation will yield multiple ancestry-associated CpGs associated with the same gene and consistent directionalities. By clustering CpGs by ancestry-specific methylation effects, we can recover patterns of such coordinated methylation change. Of note, in some cases, differential methylation can also display long-range coordination. For example, ancestry-associated CpGs found in different locations (TSS and TTS) of *SPATC1L* (see (Carrot-Zhang et al., 2020) Figures 3C and 3D) have opposite ancestry-specific effects.
    ***Note:*** As mentioned above, because the regression model uses a reference level when calculating ancestry-associated changes, one may need to rescale the mean effect sizes to zero for visualization clarity.
  d. Use patient barcodes to match normalized mRNA expression data (see key resources table, also see the following gene expression section) and plot expression against promoter methylation. One would expect to see negative associated as in Figure 3E in (Carrot-Zhang et al., 2020). See Problem 3 for potential causes for lack of correlation that may lead to low overlap between ancestry-associated differential methylation and differential expression.
  e. Validate genes with ancestry-associated regional differential methylation in whole-genome bisulfite sequencing data (see Figure 3E and 3F in (Carrot-Zhang et al., 2020)).
    **CRITICAL:** Many factors control DNA methylation. Most notably, much of the X chromosome is monoallelically methylated to balance dosage between the two sexes. Other sex-related differences have also been seen in autosomal genes such as DDX43 and OOEP (see expected results). In addition to sex, age is a major determinant of DNA methylation of CpGs of specific sequence context in late-replicating DNA and polycomb targeted CpG islands (gain of DNA methylation). Tissue-specific DNA methylation also causes tissues of different cell composition to have shifts in average DNA methylation readouts. Therefore, it is critical to include sex, age, and tumor purity or other cell type estimates as explicit covariates in the model searching for ancestry-associated DNA methylation.
    **CRITICAL:** Sample size and residual tumor heterogeneity affect the ability to find statistically significant ancestry associations (see power analysis in the quantification and statistical analysis section)
  f. Use a Wilcoxon signed-rank test to test whether a local ancestry Z-score is associated with ancestry-specific DNA methylation level. (see Figure S7D, E of (Carrot-Zhang et al., 2020) for an expected result)

### Association of ancestry with mRNA

**Timing: 2.5h**

**Timing: 1 h for step 19**

**Timing: 1.5 h for step 20**

19. Retrieving and filtering TCGA Data
  a. Download the pan-cancer mRNA data matrix (see key resources table) and exclude ancestry-admixed samples (20%<non-EUR ancestry<80%).
  b. Code .txt file with samples as rows and columns as variables (e.g., cancer type, subtype, ancestry, batch). Batch information can be extracted from the plate ID portion of the TCGA barcode.
    ***Note:*** To account for subtype-specific gene expression, the tumor type and subtype must be merged into a single variable (e.g., BRCA.Basal) in pan-cancer analyses. Only subtype (where applicable) is used in cancer-specific analyses. If subtype is not taken into account, subtype-specific gene expression may dominate ancestry associations.
  c. Code ancestry calls, such that EUR ancestry is 0 and EAS or AFR ancestry is 1.
  d. Sort the mRNA matrix so that it matches the sample and order from the preceding step.
  e. Log2-transform the gene expression matrix and filter for genes expressed in >80% of samples.
    ***Note:*** we excluded genes that show a lack of expression (zero read count) in over 20% of samples since zero read count is highly unlikely in practice and indicates low quantification. We excluded 4,262 genes, leaving 16,269 genes in the final matrix.
  f. Transpose the mRNA expression data matrix so that samples are in rows and genes are in columns (Figure 1A Bottom).

20. Identification of ancestry associated genes
  a. Perform two independent linear regressions on the gene expression data, correcting for tumor type/subtype and batch (use glm function in R):
    i. Reduced (without ancestry): [mRNA expression] ~ [tumor type / subtype] + [batch]
    ii. Full (ancestry): [mRNA expression] ~ [ancestry] + [tumor type / subtype] + [batch]
  b. For each gene, perform an ANOVA comparing the reduced model and full model's coefficients and output the beta-statistics and p-values. In R, one can use *anova(full, reduced, test="F")*
  c. Use the Benjamini-Hochberg FDR adjustment on ANOVA p-values to correct for multiple comparisons.
  d. Identify ancestry-associated genes by FDR<0.001.
    ***Note:*** we used 0.001, a stringent FDR cutoff, to focus on sizeable effect-size ancestry association discoveries (see Problem 2 for discussion)
  e. Visualize genes reaching significance using heatmaps or box plots (see Figures 4E and 4F in (Carrot-Zhang et al., 2020)).
  f. The same analysis is also performed on a per-tumor type basis as above.

***Note:*** This procedure was performed independently for AFR vs. EUR and EAS vs. EUR. After generating the output files, it is useful to spot-check results for known genes associated with the variables to ensure the sample file was coded correctly and matched the gene expression matrix. For example, one can plot median expression over ancestry for *GSTM1* (Li et al., 2015), *CRYBB2* (Huo et al., 2017), *PPIL2*, and *FBLL1* (also see (Carrot-Zhang et al., 2020) Table S4 for a list of identified ancestry-associated differentially expressed genes)

### Association of ancestry with miRNA

**Timing: 2 h**

21. Preprocess input reference mature strand data
  a. Obtain TCGA miRNA mature strand, batch-corrected, normalized reads-per-million (RPM) data (Chu et al., 2015) (see key resources table).
  b. Log2-transform RPM values, using y = log2(x+1).

***Note:*** These data include RPM expression levels of 743 miRNA mature strands (miRs) for 10,824 TCGA samples, batch-corrected to enable pan-cancer analyses. The consolidated dataset, for which both miRNA expression data and ancestry calls were available, included 8,180 samples across 32 tumor types. No miRNA sequence data were available for GBM, a pilot-phase TCGA project that used microarrays rather than sequencing. Among the 32 tumor types, 12 had subtype annotations. If batch corrected, pan-cancer atlas miRNA data are used, set negative miRNA reads-per-million (RPM) values to 0.

22. Quantify ancestry associations for mature strands
  a. Apply a linear regression model with a binary design matrix based on the subtype calls as predictors to explain the normalized RPM expression of mature strands across the samples of each tumor type and subtype.
  b. Perform Wilcoxon rank-sum tests between each pair of ancestry groups on the output of this model, applying the following pre-filtering criteria:
    i. The sample size of each group to be tested should be greater than or equal to five.
    ii. The coefficient-of-variation across the expression levels of the union of both sample groups should be 0.1 or larger.
    iii. For the union of samples of both groups, at least five samples had an RPM value of 25 or higher (4.7 in log2 space).
    iv. miRNAs that were flagged as having ancestry-specific SNPs (identified in somatic mutation association above) were discarded. See below for more details about the ancestry-specific SNPs.

**CRITICAL:** In TCGA miRNA-seq data, only reads aligned with exact matches were counted towards expression (Chu et al., 2015). Given this, TCGA samples with SNPs within mature strands will report artificially low (or zero) expression levels for these strands. Ancestry-specific SNPs in miRNAs will thus lead to spurious relationships of differential miRNA expression between ancestry groups. We, therefore, discarded mature strands with ancestry-specific SNPs. We merged miRNA annotations from miRBase (v21, released June 2014) with 1000 Genomes Phase 3 information, which contains ancestry-specific SNP allele frequencies. We called a SNP “ancestry-specific” if the difference between its maximum and minimum ancestry-specific allele frequencies among the five super-populations (AFR, AMR, EAS, EUR, SAS) was 0.25 or larger. Table S6 in (Carrot-Zhang et al., 2020) lists the SNPs in miRNAs along with their ancestry-specific allele frequencies.

23. Compile a general miRNA-host-gene data resource
  a. Download a GFF file giving miRNA annotations from miRBase v22.1 (see key resources table). Read this into R using the rtracklayer package’s readGFF function. Extract records for primary transcripts (type == “miRNA_primary_transcript”) and for mature strands (type == "miRNA").
  b. Download a GFF file giving gene and transcript annotations from Ensembl (see key resources table). Read this into R with the readGFF function. Extract (base-R subset or dplyr filter) records for genes that have ENSGs.
  c. Identify hosted miRs and host genes by seeking overlap of gene and miR transcripts above (see Problem 4). In R, one can find overlap using the GRanges mergeByOverlap function (Lawrence et al., 2013), i.e.,
    i. Apply the iRanges mergeByOverlap function to the miR and gene GRanges objects. It is required that gene and miR be on the *same strand* (ignore.strand=FALSE).
    ii. Identify miRs that are overlapped by a gene but are on the *opposite* strand from the gene, using mergeByOverlap() while ignoring strand, then taking the difference between these miRs and the same-strand hosted miRs calculated above.
    iii. Identify *intergenic* miRs as the difference between all miRBase miRs and the ignore-strand miRs calculated above.
  d. Seed sequence families
    i. Download reference miRBase mature strand sequences as ‘mature.fa’ from miRbase (see key resources table).
    ii. Reorganize the file so that each mature strand has only one row in the file. Read the new file into R.
    iii. Isolate bases 2 to 8 from the 5**′** end of each sequence, in R, using substr(sequence, 2, 8).
    iv. Tabulate distinct seed sequences and which miRNA's 5**′** or 3**′** mature strand contains each seed sequence. Associate seed sequences with miRs. See notes below on isomiRs and miRNA targeting.
24. Calculate expression correlations between miRNAs and host genes
  a. Download pan-cancer, batch-corrected, normalized RSEM GRCh37 expression data for 20531 RSEM genes and 743 expressed reference miRBase mature strands (Hoadley et al., 2018) (see key resources table).
  b. For a cancer type, given the host gene/hosted miRNAs (calculated for GRCh38 annotations, above), calculate distributions of Spearman’s correlations between RPMs for hosted a miR and RSEM normalized expression for the miR’s host gene (see Problem 5).

**CRITICAL: Reference genome assembly and gene annotations**. The latest Ensembl gene annotations are based on the recent GRCh38 assembly, but TCGA and PanCancer data are GRCh37/hg19. While we recognized that the GRCh37/hg19 TCGA RSEM gene expression data would be unavailable for some Ensembl v94/GRCh38 genes, we prioritized using near-current miRNA annotations and biotypes (see (Carrot-Zhang et al., 2020) Figure S6C) so that we would have the most up-to-date information on host genes, and noncoding polycistrons. The GRCh38-based annotation/overlap resource supported calculating correlations for 203 host genes and 331 hosted miRs (see (Carrot-Zhang et al., 2020) Figures 5E, 5F and S6F, S6K).

25. Visualize ancestry-associated miR-gene targeting
  a. To generate the UCSC hg19 screenshots, we read Table S2 from Marsico et al. 2013 (Marsico et al., 2013) into R and wrote out a zero-based BED-format file for the TSS coordinates and a bedGraph file for the TSS probabilities. We loaded both files into the UCSC hg19 genome browser as custom tracks, viewed appropriate locations, and exported PDF images of specific locations. For hsa-mir-9-3 (Figure S6J of (Carrot-Zhang et al., 2020)), we noted that the hg38 UCSC genome browser showed more MIR9-3HG transcripts than the UCSC hg19 browser, so we also downloaded a PDF of the hg38 view (though this view did not show the hg19 miRNA TSS information).
  b. To add the read pileup graphics, we went to each miRNA’s miRBase v22.1 web page and copied the read pileup graphic (e.g., http://www.mirbase.org/cgi-bin/mirna_entry.pl?acc=MI0000466 for hsa-mir-9-1). To get MIMAT mature strand IDs and reference sequences (i.e., strand sequences that showed no isomiR variation), we clicked on the miRBase ‘Get sequence’ button for a mature strand.

**CRITICAL: miRNA families.** Some miRNAs are members of miRNA families in multiple genomic locations (e.g., hsa-mir-9-1, 9-2, 9-3 (Gurtan and Sharp, 2013)). Members of such families can have identical reference miRBase mature strand sequences. The data-generating process for TCGA miRNA-seq data (Chu et al., 2015) was not designed to quantify the relative contributions of multiple locations to a mature strand's overall RPM expression level. While for some analyses (e.g., gene targeting and miR-gene expression correlations, differential miR abundance between subtypes), the location/source of a mature strand may be less critical, care should be taken in analyses that involve specific genomic locations. Such analyses include the effect of copy number alterations or DNA methylation on the expression level of a miRNA stem-loop, mature strand, or isomiR.

***Note:* isomiR variation and targeting.** A multi-step biogenesis process generates mature strands. The ‘trimmed’ locations of 5**′** and 3**′** ends of a mature strand may differ from the locations given for a reference miRBase strand (Guo and Liang, 2016) (see read pileup profiles in (Carrot-Zhang et al., 2020) Figure 5D and S6H-J). IsomiR variation at 5**′** and 3**′** ends of a mature strand may influence which RNAs are canonically and non-canonically targeted. IsomiR variation is available in both the GDC’s legacy GRCh37 miRNA-seq data generated for TCGA (Chu et al., 2015) and in the harmonized GRCh38 miRNA-seq data (Gao et al., 2019). However, in all TCGA publications and the batch-corrected pan-cancer miRNA-seq data, all mature strands were treated as ideal, reference miRBase strands.

### Integrated ancestry-associated pathways

**Timing: 4 h**

**Timing: 1 h for step 26**

**Timing: 1 h for step 27**

**Timing: 1 h for step 28**

**Timing: 1 h for step 29**

26. Retrieve and preprocess pathway activity data
  a. Download and read in the ancestry-admixture file, subtype annotation file, and the PARADIGM Integrated Pathway Level (IPL) data matrix (see key resources table)
  b. Create input files for association analysis
    i. Create a merged ancestry/subtype annotation file with samples as rows and variables (e.g., ancestry and subtype annotation) as columns
    ii. Create a PARADIGM pathway matrix file with samples as columns and pathway features as rows
  c. Filter and sort the above two input files for overlapping samples (rows of the merged ancestry/subtype file and columns of the IPL data file) by patient ID so that they have matching order
  d. Exclude ancestry-admixed samples (20%<non-EUR ancestry<80%)

***Note:*** The PARADIGM algorithm integrated platform-corrected expression, gene-level copy number, and pathway interaction data and was used to infer activities of ~19K pathway features (Data S1) for 9829 TCGA Pan-Cancer samples across 33 cancer types. The inferred activities, termed integrated pathway levels (IPLs), reflect the log-likelihood of the probability that a given feature is activated (vs. inactivated). After the above-described filtering steps, 9046 samples with PARADIGM IPL data were available for association analysis.

27. Identification of ancestry associated pathway features
  a. Identify tumor types with more than five patients of non-EUR ancestry for cancer type-specific analysis
  b. For each cancer type, filter pathway features by the following minimum variation criteria:
    i. >1 sample has abs(IPL) > 0.05
    ii. >10% of samples have non-zero IPLs
    iii. Cross-sample standard deviation of IPLs > 0.05
  c. If the cancer type is annotated with subtypes, first regress IPLs in each sample on its subtype membership. Use the residual IPLs as the IPL of each sample in the following analysis.
    i. Apply a linear regression model with a binary design matrix based on the subtype calls as predictors
    ii. Calculate the residuals from the regression as IPLs “not explained” by subtype
  d. Compare EUR and each non-EUR ancestry group using 1) t-test and 2) Wilcoxon Rank sum test.
  e. Conduct both tests within
    i. each cancer type without subtype adjustment
    ii. each cancer type with subtype adjustment
    iii. each cancer subtype
  f. Apply Benjamini-Hochberg FDR correction to all the p-values.
  g. Identify pathway feature that meets the following criteria as significantly associated with ancestry
    i. FDR corrected p < 0.05 from *both* tests (t-test and Wilcoxon rank-sum test)
    ii. abs(difference in mean IPLs) > 0.05

***Note:*** Among the 33 tumor types, 24 have ≥5 patients of non-European ancestry and were considered in the analysis. 10 of the 24 included tumor types have subtype annotation, and the subtype-adjusted and within-subtype analyses (described in steps 27b–27d) were performed.

28. Assess interconnectivity within the pathway structure to identify key regulatory nodes among ancestry associated features
  a. Download and read in the pathway interaction dictionary containing ~45K interactions linking pathway features within a superimposed pathway structure (key resources table, Data S2)
    ***Note:*** Each line in the pathway structure file is an interaction between an (upstream) parent node in the first data column and its downstream (child) node in the third data column. The middle column denotes the type of interaction. Interactions can be of the following 6 types: "-t>" = transcriptional activation ; "-t|" = transcription repression ; "-a>" = activation ; "-a|" = repression ; "component>" = component of a complex ; "member>" = member of a family.
  b. For each within tumor type, subtype adjusted, and within subtype comparison, identify ancestry-associated pathway features that are the parent nodes in >10 regulatory interactions (i.e., -a>, -a|, -t> or -t|) within the network structure as key regulatory nodes.
  c. Select the comparisons that yielded key regulatory nodes
  d. Obtain the differences in means of the key regulatory nodes for selected comparisons (already calculated in step 27g of identification of ancestry associated features)
  e. Merge differences in means into a single data matrix for display (see Figure 7A of (Carrot-Zhang et al., 2020) for an expected result)

***Note:*** This analysis required >10 regulatory interactions to be deemed a key regulatory node for a feature. This criterion can be modulated (e.g., lowered to identify more regulatory pathway features). For instance, only 143 of the ~19K pathway features have > 10 downstream targets and have the potential of being identified as a key regulatory node in our analysis. However, if one were to require a lower number of downstream targets (e.g.,> 5), 376 features would potentially be identified.

29. Identify over-represented known cancer pathways and driver genes among ancestry associated features
  a.Read in pathway feature dictionary for ~19K pathway features within the superimposed pathway structure (see key resources table)
    ***Note:*** Each line in the pathway structure file is a pathway feature and has two columns containing the feature name and feature type. The dictionary uses the term 'protein' instead of 'gene' (as different isoforms of the same gene may exist as separate features in the SuperPathway)
  b. Download the following gene sets (see key resources table)
    i. cancer driver gene set (Table S1 of (Bailey et al., 2018))
    ii. curated cancer pathway gene set (Table S3 of (Sanchez-Vega et al., 2018))
    iii. curated DNA damage repair gene set (Table S1 of (Knijnenburg et al., 2018))
  c. Create a single merged .gmt file from the three gene sets above as an input to the enrichment analysis
  d. For each comparison, use a hypergeometric test to identify over-represented gene sets in the merged input file based on the following counts (over-representation is assessed only if the odds ratio is > 2):
    i. Number of cancer pathway/driver genes identified as having ancestry-associated IPLs
    ii. Number of cancer pathway/driver genes that can be mapped to the pathway feature dictionary
    iii. Number of 'protein' features identified as having ancestry-associated IPLs
    iv. Number of 'protein' features in the pathway feature dictionary
  e. Apply the Benjamini-Hochberg FDR correction to the hypergeometric test p values
  f. Identify ancestry-associated pathways as those with FDR-corrected p values < 0.05 in the subtype-adjusted analysis (see Figure 7D of (Carrot-Zhang et al., 2020) for expected result)

***Note:*** The pathway feature and pathway interaction dictionaries are used to define the superimposed pathway structure used as input to the PARADIGM algorithm to infer pathway activities. It includes pathway interaction data from the NCIPID, Biocarta, and Reactome databases. The over-representation analysis may be a complementary approach to the key regulatory node analysis. It allows the identification of enriched pathways where no single feature has a high degree of interconnectivity (or downstream targets), which may otherwise be missed.

## Expected outcomes

### Ancestry-associated somatic alteration

See (Carrot-Zhang et al., 2020) Figure 2 for expected outcomes of ancestry-associated somatic alterations.

### Ancestry-associated DNA methylation

See (Carrot-Zhang et al., 2020) Figure 3 for expected outcomes of ancestry-associated DNA methylation analysis.

**Figure 2:**
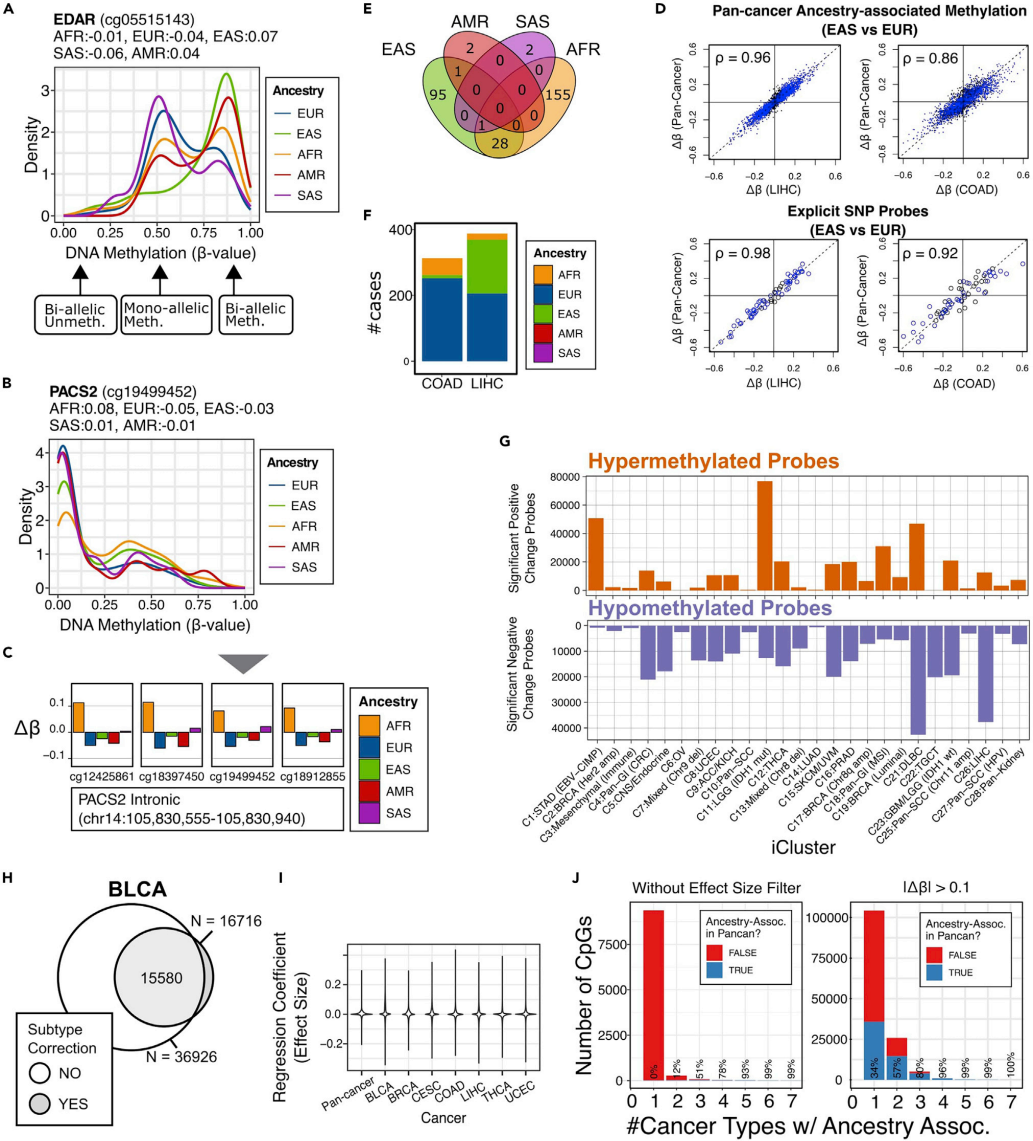
Expected outcomes (A) Distribution of ancestry-associated DNA methylation for EDAR. Slope coefficients (Δ*β*) are labeled on top of the plot. (B) Distribution of ancestry-associated DNA methylation for PACS2. (C) The slope coefficients are plotted for multiple CpGs (ordered by genomic coordinates) for PACS2. (D) Comparison of pan-cancer ancestry-association (Y-axis) with cancer-type-specific ancestry-association (X-axis, showing LIHC on the left and COAD on the right). (E) Venn diagram showing the number of significant associations of non-EUR ancestry group with EUR. (F) Bar plot for the number of non-EUR cases for COAD and LIHC. (G) Slope coefficients associated with iCluster memberships. (H) The overlap between subtype-corrected ancestry association and uncorrected inference on BLCA. (I) The effect size distribution of Δ*β* in each cancer type. (J) Distribution of cancer type-specific ancestry association in the number of cancer types where this association is found (X-axis). The overlap with pan-cancer analysis is labeled on top of each bar. The left panel shows the distribution without the effect size filter. The right panel corresponds to after filtering |Δβ|>0.1.

A bona fide ancestry association should show a clear difference between ancestries. For example, if one were to plot DNA methylation from different ancestry groups, one should see a clear distinction in the distribution of the DNA methylation level (Figure 2A). DNA methylation is categorical in a cell, displaying three modes representing bi-allelic unmethylation, mono-allelic methylation, and bi-allelic methylation. For example, for the ancestry-associated methylation associated with the EDAR gene, EAS and AMR have more bi-allelically methylated subjects than SAS and EUR (Figure 2A). This disparity can be visualized by plotting the scaled slope coefficients from the regression (Figures 2A and 2B, labeled on the top of each figure). One can see that neighboring CpGs show coordinated ancestry association with a similar pattern in the regression coefficients. It should also be noted that cancer types with better representation of the corresponding ancestry group have greater power of detecting ancestry-associated changes (showing differential DNA methylation). Plotting the EAS-EUR-specific differential methylation in different cancer types indicate that cancer types with more EAS subjects (showing LIHC, Figures 2D and 2F) can detect this ancestry association with greater confidence than cancer types with fewer EAS subjects (showing COAD, Figures 2D and 2F). More ancestry-associated differences are found in AFR and EAS than in AMR and SAS, consistent with the relative abundance of these non-EUR groups in TCGA (Figure 2E). Cancer type and subtype should be driving most DNA methylation variation (Figure 2G). Accounting cancer subtype could significantly reduce the number of ancestry association, likely improving detection accuracy (Figure 2H). It should also be expected that most ancestry-associated differences are of small effect sizes (Figure 2I). Most small-effect-size ancestry association is specific to one or two cancer types and cannot be repeated in pan-cancer analysis, suggesting either cancer specificity of these finding or low robustness of the discovery (Figure 2J, left). Filtering effect size can significantly increase the rate of validation (Figure 2J, right). One should also expect validation from external data sets. For example, in our original publication (Carrot-Zhang et al., 2020), we validated our ancestry-associated differential DNA methylation finding against four different data sets, covering overlapping or completely different ancestry backgrounds (see Figures 3F, 3G, and Figure S4 of (Carrot-Zhang et al., 2020)).

### Ancestry-associated mRNA analysis

See (Carrot-Zhang et al., 2020) Figure 4 for expected outcomes of the ancestry-associated mRNA expression analysis.

### Ancestry-associated miRNA analysis

See (Carrot-Zhang et al., 2020) Figure 5 for expected outcomes of the ancestry-associated miRNA expression analysis.

Figure 5D of (Carrot-Zhang et al., 2020) is a UCSC screenshot of Marsico et al., 2013 TSSs for hsa-mir-628, hosted by *CCPG1*. The figure also shows a miRBase v22.1 read pileup profile, which indicates that, for this miRNA, we expect the 5p strand to be more highly expressed than the 3p strand. miRBase read pileups also suggest the level of 5**′** and 3**′** isomiR variation to expect for each mature strand. Figure S6H-J of (Carrot-Zhang et al., 2020) shows similar information for the three members of the miR-9 family, miR-9-1, -2, and -3, for which the 5p strand and 3p strand have identical reference sequences in all three locations.

In compiling the data resource, we sought to support calculating miR-host gene correlations and offer contextual information to a researcher who has identified one or more miRs associated with ancestry in a disease. Such a researcher may want to infer how a miR may influence the disease biology and prioritize miRs for additional work. The data resource we compiled lists factors that may be useful to consider in interpreting and prioritizing ((Carrot-Zhang et al., 2020) Figure S6B). Below, we briefly discuss several such factors:

Current miRNA annotations: For the ancestry-associated mature strands in ((Carrot-Zhang et al., 2020) Figure S6A), the table in that panel notes that miR-3607-3p and 3653 have been removed from miRBase. Such removals are more likely to occur for miRNAs with large-number names (say, larger than 800, e.g., “3607”) because their mature strands will tend to be weakly expressed. Such miRNAs were added to miRBase more recently. Given this, it is useful to check whether miRNAs and mature strands that appear interesting are still considered by miRBase to be valid miRNAs. A simple way to do this is to search for a miRNA name (e.g., “hsa-mir-3607”) at miRBase; if the miRNA has been removed, miRBase will return a “Dead miRNA entry” page. An alternative resource is ‘miRNA.dead’. For brief discussions of many miRNA issues, including seed types, see TargetScan’s FAQs (http://www.targetscan.org/faqs.Release_7.html).

miRNA transcriptional start sites (TSSs): In TCGA marker papers, for location-specific calculations of the relationship between DNA methylation and miRNA expression, we used DNA methylation probes that were close to miRNA TSS’s reported from FANTOM deep CAGE sequencing, e.g., Table S2 from (Marsico et al., 2013), and (Carrot-Zhang et al., 2020) Figure S6H-J. For example, TSS predictions were helpful when a miRNA was expressed from a long poly-cistron whose TSS was some distance upstream, and in distinguishing cases in which a hosted miRNA had (or lacked) one or more TSSs that were independent of the host gene’s TSS. Alternative and updated sets of miRNA TSSs are available from more recent work (Consortium et al., 2017; Wang et al., 2020), and, for location-specific calculations, these and other resources should be carefully assessed so that an appropriate resource is used.

Polycistrons and miRNA clusters: Some miRNAs are expressed from longer noncoding polycistronic transcripts ((Carrot-Zhang et al., 2020) Figure S6B, also see (Donayo et al., 2019; Ichiyama and Dong, 2019)). Mutations within a polycistron can upset the polycistron’s bioprocessing, changing the relative abundance of the miRNAs expressed from that polycistron (Chakraborty et al., 2012). For work with polycistrons, it is helpful to use up-to-date information on transcripts.

### Ancestry-associated pathway

See (Carrot-Zhang et al., 2020) Figure 7 for expected outcomes of the ancestry-associated pathway analysis.

## Quantification and statistical analysis

### Power Analysis

**Timing: 1 h**

To explore the extent of false negatives, we performed a power analysis for all molecular correlates. The following protocol details the simulation procedure to estimate the power to detect ancestry-associated somatic mutations.

1. Read in TCGA ancestry assignment and cancer subtype (key resources table).
2. Assign the frequency of somatic mutation (intercept) and the odds ratio (slopes).
3. For each mutation with a specific frequency at a different odds ratio, simulate 1000 times generalized linear regression between somatic mutation and ancestry assignment, controlling for the subtype. Power is defined as the percentage of tests returned with a p-value less than 5e-05 (assuming testing 20,000 genes followed by Bonferroni correction).
4. Plot mutation frequency with different odds ratio against power.
5. The following protocol details the simulation procedure to estimate the power to detect ancestry-associated differential DNA methylation.
  a. Simulate DNA methylation levels for each CpG included on the HM450 platform using a beta distribution Beta(1,5) for gender-specific methylation differences and a normal distribution of N(0.1,0.2) (capped at 0, and 1) for subtype-specific methylation differences.
  b. For each given methylation level, compute the DNA methylation readout as a binomial distribution Binom(N=40), modeling the average bead number for each probe in the HM450 microarray.
  c. Simulate methylation readouts for all HM450 CpGs 1000 times
  d. Perform the same regression analysis for each simulation using the real ancestry annotation.
  e. Compute the statistical power as the fraction of inferred significant ancestry-specific differences over all the simulated methylation differences. Plot power against simulated methylation differences.
  f. Repeat that process for pan-cancer and each cancer type.

**CRITICAL:** The TCGA dataset is not balanced with equal representation of patients of different gender, subtype, and ancestry backgrounds. To faithfully reflect the dataset's limitations, power analyses should maintain the population structure and only permutate the ancestry label. It is expected that greater simulated methylation differences and more samples, especially of the less-represented ancestry, will result in greater power to detecting ancestry-associated differential methylation. For example, EAS-EUR differences are better captured in LIHC, where Asians are well-represented, than in BRCA, where they are not. Conversely, AFR-EUR differences show the opposite effect because African ancestry is better represented in the TCGA BRCA cohort (Carrot-Zhang et al. (Carrot-Zhang et al., 2020) Figure S1).

***Note:*** The power calculation for DNA methylation can be adapted to analyze the statistical power of detecting other continuous ancestry-associated molecular correlates including mRNA and miRNA.

## Limitations

### Under-representation of non-European samples

In TCGA, non-European samples are underrepresented except for a few cancer types (see Carrot-Zhang *et al.* (Carrot-Zhang et al., 2020) Figure 1B). This leads to a lack of statistical power of detecting ancestry associations, particularly those correlated with small effect size and those associated with the underrepresented ancestry groups (see Carrot-Zhang *et al.* Figure S1). Therefore, we focused on pan-cancer ancestry association and cancer type-specific association only in eight cancer types with sizable non-European patients. While this limits our capability to detect more subtle association in underrepresented ancestry groups, the methodology can be used to analyze more balanced cohorts and cohorts with better coverage of the non-European ancestries.

### Tumor purity

We used a purity threshold in performing our analysis. However, the residual samples may still be influenced by purity. This influence is particularly relevant to epigenetic and transcriptomic correlations. Different cell components carry very distinct profiles, and bulk estimates contain convoluted signals from all the constituent cell components. Failure in accounting for tumor purity may result in an inability to distinguish alternative interpretation using cell composition shift from tumor-intrinsic molecular aberrations. It also increases the chance of being confounded by hidden differences in sample acquisition and processing. In some models (Table 1), we included tumor purity explicitly as a linear confounder in our model. The final solution requires comprehensive deconvolution of cell components in each sample.

**Table 1:** Regression models for detecting ancestry-associated molecular correlates

Alternative hypothesis	Method	Example model
Somatic alteration is associated with local ancestry	Logistic Regression	somatic alteration ~ local ancestry + percentage of EUR ancestry + percentage of AFR ancestry
Ancestry is associated with arm-level SCNA	Logistic Regression	ancestry ~ arm-level SCNA + aneuploidy + genome doubling + age + gender + subtype
Ancestry is associated with TMB, aneuploidy, and IMS	Logistic Regression	ancestry ~ aneuploidy + TMB + IMS + age + gender + subtype
DNA methylation is associated with ancestry	Linear Regression	DNA methylation level (*β* value) ~ ancestry + tumor purity + age + gender + subtype
mRNA expression is associated with ancestry	Linear Regression	log2(RPM) ~ ancestry + TCGA plate number + subtype
miRNA expression is associated with ancestry	Linear Regression	log2(RPM+1) ~ ancestry + subtype
Pathway activity is associated with ancestry	Linear Regression	IPL ~ ancestry + subtype

RPM: Read Per Million. IPL: Integrated Pathway Level.

### Survival bias

Studying ancestry-associations of molecular correlates in cancer cohorts may be subject to survival bias---when the genetic background specific to an ancestry group provides protection or predisposition of cancer, these populations will be under or over-represented in the corresponding cancer types. This bias may result in diminished power in detecting associations in cancer types where the cancer incidence in that ancestry group is rare.

### Null model

Our analysis assumes normal error distributions in performing linear regressions on ancestry-associated molecular correlates. This assumption holds valid when the sample number is large. One may need to take caution applying our analysis framework to smaller datasets and use alternative approaches such as using beta regression or empirical post-processing of p-values.

### miRNA seed sequence families

While we report no gene targeting information in our public data resource, we report seed sequence families. The seed sequence of an expressed mature strand, or isomiR, consists of 5**′** bases 2 to 7 or 8 (seed types are described at the TargetScan FAQ, see key resources table). The seed sequence is accepted as an important influence in targeting (Agarwal et al., 2015) (but see isomiRs, below). Because the same seed sequence can be present in more than one mature strand, mature strands can be grouped into sets or ‘families’ that share a seed sequence. Considering the potential biological influence of an ancestry-associated mature strand, it is worth checking whether the mature strand shares its seed sequence with other family members.

### miRNA targeting: Choice of annotation and their limitations

Resources that offer experimentally validated targeting (Chou et al., 2017) will tend to be biased by which mature strands and genes have been more (vs. less) thoroughly characterized. Computational target predictions will be free from such experimental biases, but their accuracy will be constrained by uncertain rates of false positives and false negatives. Besides, because of ‘sponging’ by competitive endogenous RNAs (ceRNAs), miRs that are more weakly expressed are less likely to influence biology (Chiu et al., 2018; Mullokandov et al., 2012; Thomson and Dinger, 2016); miR-target binding affinity will also interact with the concentrations of mature strands/isomiRs and transcripts (Bosson et al., 2014). ceRNAs will include a range of coding and noncoding gene biotypes ((Carrot-Zhang et al., 2020) Figure S6C). While seed-based miRNA targeting data is available for lncRNAs (Karagkouni et al., 2019), we note that TargetScan (in its FAQs) declines to offer target predictions on lncRNAs because many lncRNAs are mostly nuclear, while mature strands will be active in the cytoplasm (See key resources table).

### Ancestry group definition

Some ancestry groups are better defined than others. Some ancestry groups might be considered admixed in themselves. For example, the AMR group is largely composed of individuals of Latin American origin with admixed EUR and Native American backgrounds. Likewise, SAS is an admixed group. One could exclude admixed groups from the analysis. In our data set, the AMR and SAS groups are relatively rare, leading to a lack of power in detecting AMR and SAS-specific ancestry association. This lack of power is consistent with discovering only 2 AMR and 2 SAS ancestry-associated CpGs from our analysis (see expected outcomes and Figure 2E). These findings warrant further follow-up in cohort with better coverage of these ancestry populations.

## Troubleshooting

### Problem 1 (step 9)

Lack of ancestry association in cancer types with non-European ancestry underrepresented

### Potential solution

TCGA cohort does not cover different ancestry groups equally. Non-European ancestry is particularly underrepresented in certain cancer types such as ACC, MESO, and UVM. This bias makes it hard to distinguish cancer type association from ancestry association. It also hinders the detection of cancer-type-specific ancestry associations. To address this issue, one should exclude these cancer types while studying pan-cancer ancestry association.

### Problem 2 (steps 17d and 20)

One may discover many ancestry associations with small effect sizes (Figure 2I). Small effect size can indicate minor ancestry association or residual heterogeneity in cancer types and subtypes. The biological significance of the small change in molecular correlates is not clear. For example, DNA methylation is categorical, taking 0, 0.5, and 1 in methylation fraction for each diploid cell. An ancestry-associated difference of a small fraction (e.g., <0.1) can suggest high heterogeneity within each ancestry group.

### Potential solution

One can apply a fixed-threshold filter to remove ancestry-associated correlates of small effect size. For the best threshold, one could use an external or pan-cancer set for validation. Our result suggests that a high effect-size cancer type-specific ancestry association is more likely to be validated in the pan-cancer analysis. (Figure 2J). An alternative approach would be to use a more stringent FDR threshold. For example, in identifying ancestry-associated mRNA, we used 0.001 as our significance threshold. One can also use simulation to estimate the type-II error rate.

### Problem 3 (step 18d)

A low overlap between ancestry-associated differential methylation and differential expression (Figure S7F in (Carrot-Zhang et al., 2020)).

### Potential solution

CpG methylation might not be indicative of transcript expression. Typically, DNA methylation is associated with transcriptional silencing only at gene promoters and cis-regulatory elements. These transcription-associated CpG can be hard to identify and vary by cell type context. Besides, DNA methylation does not fully determine gene expression. Other activating and repressive epigenetic modifications can co-regulate gene transcription. Complementary epigenetic silencing can confound the absence of gene expression. For example, polycomb-repressive complex (PRC)-mediated suppression of gene expression is a more reversible parallel mechanism of epigenetic silencing (Schuettengruber et al., 2017). It typically takes place in the unmethylated genomic territory. On the other hand, switched epigenetic control does not always escalate to a change in gene expression. Checking orthogonal epigenetic modifications and inspecting a gene’s regulome holistically may reveal discrepancies in ancestry association with DNA methylation and gene expression.

### Problem 4 (step 23c)

Ancestry-associated miRNA has too many target genes. It is hard to prioritize the most disease-relevant targets.

### Potential solution

Typically, miRNA targeting can lead to a targeted RNA transcript being degraded; for coding genes, targeting can also interfere with protein translation (Gurtan and Sharp, 2013). For ranking potential miR target genes, it is helpful when computational target predictions are accompanied by experimentally-based ‘scores’ that reflect how strongly targeting should reduce an mRNA’s concentration. Certain computational target predictions offer both predictions and scores; e.g., TargetScan v7.x (Agarwal et al., 2015) and miRmap (Vejnar and Zdobnov, 2012).

### Problem 5 (step 24b)

A lack of correlation between ancestry-associated miRNA transcription and host gene expression (Figure 5E in (Carrot-Zhang et al., 2020)).

### Potential solution

One can check whether the miRNA and host gene are transcribed on the same strand. Moreover, if the miRNA and host gene transcription happen on different strands, the miRNA might not be hosted by the host gene. If the miRNA has its promoter, it can also cause de-coupling of the miRNA expression and the host gene. The miRNA transcription will then be driven by the promoter independent of the host gene transcription.

## Resource availability

### Lead contact

Further information and requests for resources and reagents should be directed to and fulfilled by the lead contact, [Andrew D. Cherniack] (achernia@broadinstitute.org).

### Materials availability

This study did not generate new unique reagents.

### Data and code availability

Data used and generated are listed in the key resources table, and the GDC Publication Website (https://gdc.cancer.gov/about-data/publications/CCG-AIM-2020).

## Supplemental information

Data S1. PARADIGM integrative pathway level feature definition (step 26)

Data S2. PARADIGM integrative pathway level interaction dictionary (step 28)

## Footnotes

Supplemental information can be found online at https://doi.org/10.1016/j.xpro.2021.100483.
